# Strong Dependence of Surface Enhanced Raman Scattering on Structure of Graphene Oxide Film

**DOI:** 10.3390/ma11071199

**Published:** 2018-07-12

**Authors:** Ling Wang, Yan Zhang, Yongqiang Yang, Jing Zhang

**Affiliations:** 1 School of Material Engineering, Shanghai University of Engineering Science, Shanghai 201620, China; lingwangwz@163.com (L.W.); zhangjinglyy@163.com (J.Z.); 2 Jiangsu Graphene Inspection Technology Key Laboratory, Jiangsu Province Special Equipment Safety Supervision and Inspection Institute Branch of Wuxi, Wuxi 214174, China; yqyang@wxtjy.com

**Keywords:** graphene oxide, graphene, surface-enhanced Raman scattering (SERS), oxygen-containing groups, chemical enhancement, *sp*^2^ carbon domain

## Abstract

Graphene and its derivatives have been demonstrated to be good surface-enhanced Raman scattering (SERS) substrates. However, the literature offers some contrasting views on the SERS effect of graphene-based materials. Thus, understanding the mechanism of the SERS enhancement of graphene is essential for exploring its application as a SERS substrate. In this study, graphene oxide (GO) and chemically reduced graphene oxide (CRGO) films with different morphologies and structures were prepared and applied as SERS substrates to detect Raman dye molecules. The observed enhancement factors can be as large as 10~10^3^. The mechanism of SERS enhancement is discussed. It is shown that the SERS effect was independent of the adsorption of dye molecules and the surface morphologies of graphene-based films. Raman shifts are observed and are almost the same on different graphene-based films, indicating the existence of charge transfer between dye molecules and substrates. The Raman enhancement factors and sensitivities of dye molecules on different films are consistently within the *I_G_*/*I_D_* ratios of graphene-based substrates, indicating that the dramatically enhanced Raman spectra on graphene-based films are strongly dependent on the average size of *sp*^2^ carbon domain.

## 1. Introduction

Surface-enhanced Raman scattering (SERS) is a highly selective, sensitive, and harmless spectroscopic technique to detect molecules, which is widely used in bio-sensing, medical diagnosis, environmental testing and other areas [1,2,3,4,5,6]. It is well-known that the two widely accepted mechanisms of SERS are the electromagnetic enhancement mechanism (EM) and chemical enhancement mechanism (CM). The EM is related to the surface plasmonic elements of the metal particles, while also related to the surface plasmon resonance and nanoparticle morphology, structure, composition and coupling situation [7,8,9]. The CM is based on the charge transfer resonance between molecules and substrates [10,11]. These two inseparable mechanisms are usually concomitant in practice, and the contribution from CM is often much weaker than EM in the entire SERS enhancement, which leads to the big challenges in investigating the CM in SERS.

Graphene, as a two-dimensional carbon material, has drawn considerable research attention owing to its theoretically large specific surface area and excellent electronic and optical properties [12,13]. Owing to high adsorption capacity, fluorescent quenching, and Raman scattering enhancement, graphene has been demonstrated to be a SERS substrate [14,15,16]. To date, graphene, graphene oxide (GO), and graphene nanocomposites were used to enhance Raman spectra [15,17,18]. However, the literature offers some contrasting views on the SERS activity of graphene. Zhang et al. demonstrated that the intensities of the Raman signals were enhanced on the graphene surface [19]. Yu and Yin et al. found that mildly reduced graphene oxide nanosheets increased the chemical enhancement for adsorbed molecules, in comparison with mechanically exfoliated graphene [20,21]. Nam et al. reported that a highly oxidized graphene enhanced SERS activity compared to CVD (chemical vapor deposition) -grown graphene [22]. Yu et al. showed that GO resulted in a stronger Raman enhancement than reduced graphene oxide and graphene, and the Raman signals on GO increased with the number of GO layers [23]. Zhang et al. reported a clear Raman enhancement effect on the surface of monolayer graphene rather than multilayer graphene [19], and SERS effect is independent of the number of graphene layers in the six layers range [16]. Compared with a monolayer or few-layer graphene and GO, Zhang et al. demonstrated that a reduced graphene oxide paper-like film with controllable shapes and a convenient manipulation was a good substrate for SERS detection [24]. Thus, understanding the mechanism of the SERS enhancement of graphene is essential for exploring its application as a SERS substrate. In addition, it has been proved that Raman enhancement of graphene based on the CM was prominent only [19]. Graphene as a SERS substrate is helpful to better understanding the fundamental principles of SERS phenomena.

In this work, series of GO films and chemically reduced graphene oxide (CRGO) films were prepared by different drying methods. Scanning electron microscopy (SEM), Raman spectroscopy, and X-ray photoelectron spectroscopy (XPS) were used in characterizing surface morphologies, structural changes and chemical groups. By using rhodamine 6G (R6G), malachite green (MG), and crystal violet (CV) as Raman probes, we systematically investigate the SERS effect of GO and CRGO films. In combination with those results, we suggested that the size of each *sp*^2^ domain and oxygen-containing groups in graphene are responsible for the large Raman signal enhancement. GO-LN film which was subjected to liquid nitrogen and freeze-drying with high oxygen-containing groups and large average size of *sp*^2^ carbon domains has a remarkably improved Raman enhanced activity. SERS signals on GO-LN films have good linear responses to the concentration of different Raman dye molecules, which shows great potential in bio-sensing, medical diagnosis, environmental testing and other areas.

## 2. Material and Methods

### 2.1. Materials

Rhodamine 6G (R6G, 95%) was purchased from Sigma–Aldrich (St. Louis, MO, USA). Ascorbic acid (L-AA, ≥99.7%, AR), crystal violet (CV, AR) and Malachite Green (MG, AR) were all purchased from Aladdin Biochemical Technology Co., Ltd. (Shanghai, China). All aqueous solutions were prepared using ultrapure water (18.2 MΩ·cm). GO were prepared by a modified Hummer’s method, and CRGO was obtained by reducing graphene oxide with L-ascorbic acid [25].

### 2.2. Preparation of GO Films and CRGO Films

To prepare the GO or CRGO film, 50 mL of 1.0 mg/mL GO or CRGO dispersion was filtered through a mixed cellulose ester filter membrane (0.2-μm pore size, Aladdin Biochemical Technology Co., Shanghai, China) by vacuum suction. Once the filtration was completed, the fresh GO or CRGO film was immediately immersed in liquid nitrogen. After the film was completely frozen, the samples were placed in a freeze dryer for 12 h, and then they were carefully separated from the filter membrane, generating GO-LN or CRGO-LN. GO-h and CRGO-h films were prepared by drying the fresh GO and CRGO films at 40 °C for 6 h. GO-f and CRGO-f films were prepared by freezing the fresh GO and CRGO films at −20 °C for 12 h and then dried using the freeze-drying method.

### 2.3. SERS Detection

For SERS measurement, GO and CRGO films were immersed in different concentrations of Raman dye molecule (R6G, MG, or CV) for 24 h in order to reach the adsorption equilibrium. After that, the samples were rinsed with ultrapure water to remove free molecules and then dried in air. The Raman spectra were recorded using a LabRAM HR Evolution Raman spectrometer (HORIBA Scientific, HORIBA Jobin Yvon, Paris, France) with a 100× objective lens. The SERS detection was carried out with a 532-nm laser source, the laser power of 0.5 mW, and the accumulation time of 30 s.

### 2.4. Characterization

Scanning electron microscope (SEM) images were acquired on an S-3400-emission scanning electron microscope (Hitachi, Tokyo, Japan). XPS spectra were collected on an AXIS Ultra spectrometer (Kratos Analytical, Inc., Manchester, UK) with a monochromated Al *K*α source.

## 3. Results and Discussion

**Figure 1:**
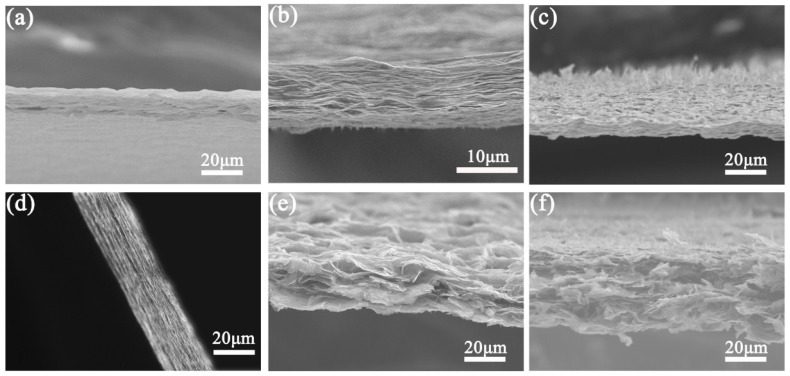
SEM cross-sectional images of (**a**) graphene oxide (GO)-h; (**b**) GO-f; (**c**) GO-LN; (**d**) chemically reduced graphene oxide (CRGO)-h; (**e**) CRGO-f; and (**f**) CRGO-LN.

The surface morphologies of as-prepared samples were elucidated by SEM. Figure 1 shows the cross-sectional SEM images of GO and CRGO films. A layered structure can be clearly observed. Compared to GO-h and CRGO-h films, GO-LN and CRGO-LN films have larger interlamellar spacing. In particular, many vertical GO sheets or CRGO sheets are shown on the surface of GO-LN or CRGO-LN film. Compared with the GO-LN film, a more open and disordered structure of the inner CRGO-LN film is observed. The difference in the morphology of the inner film is consistent with water contact angles of GO and CRGO surface [26], which originates from the hydrophilicity/hydrophobicity of GO and CRGO surface.

**Figure 2:**
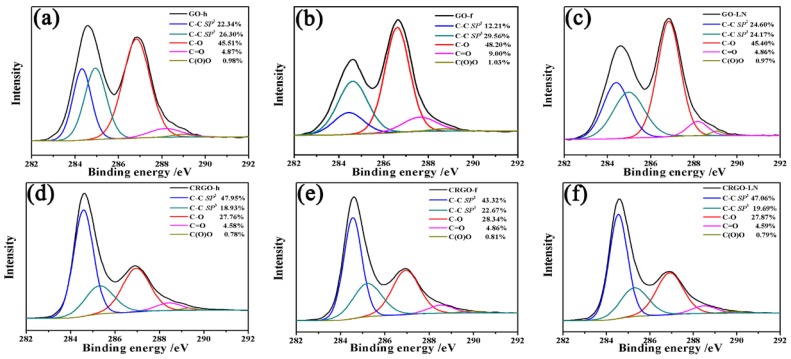
High-resolution deconvoluted C 1*s* XPS spectra of (**a**) GO-h; (**b**) GO-f; (**c**) GO-LN; (**d**) CRGO-h; (**e**) CRGO-f; and (**f**) CRGO-LN films.

In order to identify the elements’ states in materials, as-prepared samples were investigated by XPS. The high-resolution C 1*s* spectra of the GO-h, GO-f, GO-LN, CRGO-h, CRGO-f, and CRGO-LN films, including curve fits for the various carbon components, are presented in Figure 2. In addition, the percentages of functional groups and the *sp*^2^ fractions were calculated from XPS spectra for a better comparison. All of the samples have almost the same content of COOH groups, except CRGO-f. Upon the reduction, the intensities of the XPS peaks of C–O (epoxy and alkoxy) decreased rapidly, indicating that most of the epoxide and hydroxyl functional groups were removed. The total areas of the *sp*^2^ domains of CRGO films increase, which results in a remarkable increase of the conductivity (Table S1). Meanwhile, the relative peak areas of *sp*^2^ C–C in GO films and conductivity of GO films were calculated, and the component of *sp*^2^ carbon and the conductivity are both in the order of GO-LN > GO-h > GO-f (Figure 2 and Table S1), suggesting an improved degree of graphitization of GO-LN.

**Figure 3:**
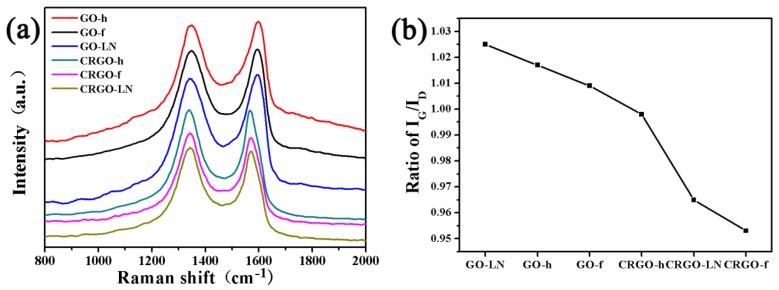
(**a**) Raman spectra of GO and CRGO films; (**b**) *I_G_*/*I_D_* ratios of GO and CRGO films.

Raman spectra were measured and are shown in Figure 3. It is clear that all of the samples exhibited a *D* band and a *G* band at approximately 1345 and 1590 cm^−1^, respectively [25]. Generally, the ratio of the *G*- and *D*- band intensity (*I_G_*/*I_D_*) in normal Raman spectra of GO and CRGO can be used to estimate the average size of *sp*^2^ carbon domain, and the higher ratio of *I_G_*/*I_D_* indicates the larger average size of *sp*^2^ carbon domain [17]. Although the total area of the *sp*^2^ domains of CRGO films increased after reduction as mentioned above, because of the increasing defective carbon domains that were generated after the removal of oxygen-containing groups, the size of each *sp*^2^ domain of CRGO decreased, which destroyed the π-conjugation of the substrate. Thus, the *I_G_*/*I_D_* ratios of CRGO films are lower than those of GO films (Figure 3b). Compared with those GO and CRGO films, GO-LN shows the largest ratio of *I_G_*/*I_D_*, indicating that partial *sp*^2^ domains were restored and the graphitic degrees of GO-LN were also improved.

**Figure 4:**
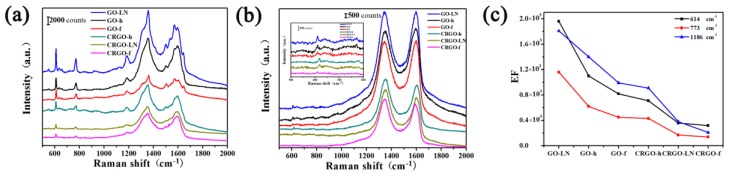
Raman spectra of R6G deposited on different films by soaking in (**a**) 1 × 10^−6^ M and (**b**) 1 × 10^−7^ M R6G solution; (**c**) Enhancement factors (EFs) for the main vibration modes of R6G (1 × 10^−6^ M) on different films.

**Figure 5:**
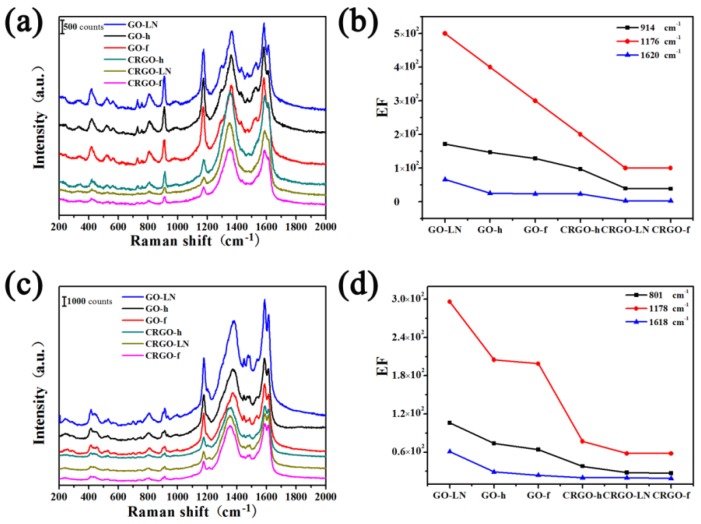
(**a**) Raman spectra and (**b**) enhancement factors (EFs) for the main vibration modes of CV deposited on different films by soaking in 1 × 10^−5^ M CV solution; (**c**) Raman spectra and (**d**) EFs for the main vibration modes of MG deposited on different films by soaking in 1 × 10^−4^ M MG solution.

To investigate the SERS effect of GO films and CRGO films, R6G, CV and MG were chosen as the target molecules. As shown in Figure 4a,b and Figure 5a,c, a series of Raman spectra of R6G, CV and MG on the surface of different graphene films can be identified definitely by the main peaks of the vibration modes. The R6G molecules exhibit the vibrational bands corresponding to a bending vibration of C_X_–C_X_–C_X_ on the X-ring at 614 cm^−1^, the C_X_–H bending vibration at 773 cm^−1^ and 1306 cm^−1^, a C_X_–Cx stretching mode at 1186 cm^−1^, and the stretching vibration of C_X_–C_X_ (X stand for xanthene rings) at 1366 cm^−1^ and 1648 cm^−1^ [27]. The peak of the CV molecule at 809 cm^−1^, 914 cm^−1^ and 1176 cm^−1^ results from the aromatic C–H bending, and the peak at 1535 cm^−1^, 1585 cm^−1^ and 1620 cm^−1^ belongs to the aromatic C–C stretching [22]. The peak of the MG molecule at 801 cm^−^^1^ and 915 cm^−^^1^ attributed to ring C–H out-of-plane bending, the peak at 1178 cm^−^^1^ belongs to ring C–H in-plane bending, and the peak at 1618 cm^−^^1^ results from ring C–C stretching [28]. The Raman signals of R6G, CV and MG on different films are stronger than those on the glass slide substrate (Figure S1), suggesting that the different graphene can be used as a substrate for Raman enhancement. At the resonant wavelength excitation of R6G and CV, 532 nm, the spectra with strong Raman signals were observed on graphene, which could be attributed to both resonant enhancement and chemical enhancement. At 532 nm excitation, which is outside the absorption range of MG, the Raman signals from the molecules are still visible, suggesting chemical enhancement exists, which results in strong signals. For better comparison, the enhancement factor (EF) values are calculated (Table S2, see the Supporting Information for the calculation details of EF values). The observed enhancement factors can be as large as 10~10^3^, indicating that observable chemical enhancement can exist. These results suggested that the Raman enhancement was based on the chemically enhanced mechanism.

It is interesting to find out that the Raman intensities and EF values of R6G on different films took the order of GO-LN > GO-h > GO-f > CRGO-h > CRGO-LN > CRGO-f, and the same trend is observed for CV and MG. According to the SEM results, both GO-LN and CRGO-LN films have vertical nanosheets on the surface. It is shown that GO-LN exhibited the largest SERS activity, while CRGO-LN resulted in a weaker Raman enhancement than GO films and CRGO-h film. Apparently, the Raman enhancement on different graphene films is not related to surface morphology. In order to explain these phenomena, the abilities of different films to adsorb R6G molecules were considered. The quantities of molecules deposited on the substrates were obtained by subtracting the amount of the left R6G in the supernatant (which determined by measuring the UV absorbance of R6G solution at 527 nm) from the total R6G added. We found that the R6G contents on the different substrates are almost the same (Table S3). This is consistent with the rhodamine B contents on different reduction degree of CRGO substrates [20]. Thus, the effect of the adsorption of R6G on SERS effect can be ignored. As is known, the sufficient oxygen-containing groups possess a strong local dipole moment that can induce a considerable local electric field under laser excitation [17]. XPS analyses show the contents of C–O (epoxide and hydroxyl groups) on GO films are much larger than those on CRGO films. Compared to CRGO films, the sufficient oxygen-containing groups could contribute to a stronger SERS effect of GO films. However, it was worthy of note that the contents of C–O on GO and CRGO films decreased in the order of GO-f > GO-h > GO-LN > CRGO-f > CRGO-LN > CRGO-h. Thus, this could not explain the different SERS effects of graphene-based materials correctly. Chemical enhancement based on charge transfer is thought of as an important factor of SERS. Herein, the peak positions of R6G, CV, and MG on different films are compared with those on glass slide substrate, respectively. From Table S2, the Raman shifts on both GO films and CRGO films are observed, confirming the strong interaction between dye molecules and substrates and proving the existence of charge transfer [15]. However, those Raman shifts are almost the same in correspondence with that reported in the literature [20], indicating that the changes in chemical compositions between GO and CRGO films have little effect on the basic interaction between the substrate and the molecule.

**Figure 6:**
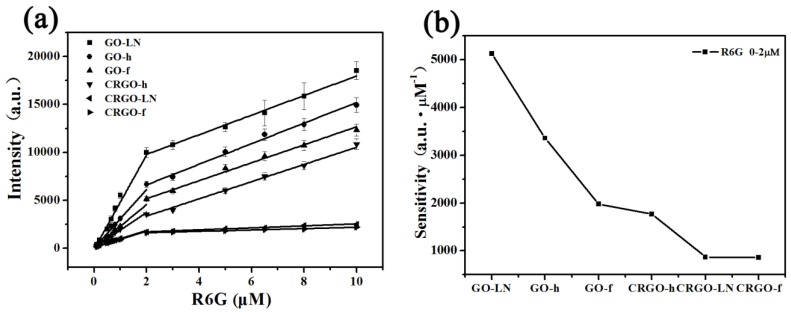
(**a**) The corresponding plots of Raman intensity of ~614 cm^−1^ peak of R6G deposited on GO and CRGO films vs. concentration; (**b**) the SERS sensitivity of R6G on GO and CRGO films in 0.1–2 × 10^−6^ M R6G region.

**Figure 7:**
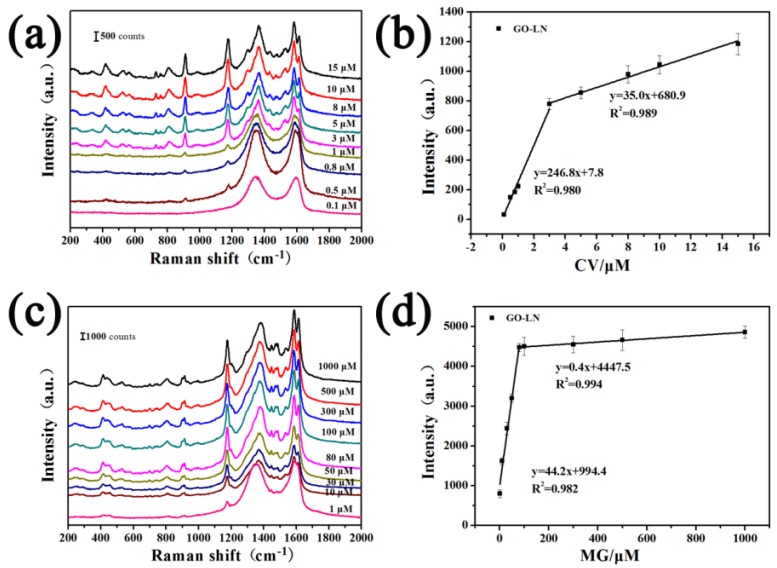
Raman spectra of (**a**) CV and (**c**) MG molecules deposited on GO-LN films by immersing in the solution with different concentrations; (**b**) the corresponding plots of Raman intensity of ~914 cm^−1^ peak of CV deposited on GO-LN vs. concentration; (**d**) the corresponding plots of Raman intensity of ~1178 cm^−1^ peak of MG deposited on GO-LN vs. concentration.

Raman spectra of R6G molecules on GO-LN films with different concentrations are shown in Figure S2. The intensities of the Raman signals of the molecules on GO-LN film decrease with a decrease in the concentration. The quantitative relation between the concentration of R6G and the intensity of characteristic Raman band at ~614 cm^−1^ on different films were plotted in Figure 6a. Every data point represents an average intensity from five measurements and the error bars reveal standard deviations within a 11.7% variation range. From the corresponding calibrated plot, a linear relationship between R6G concentration and Raman intensities is observed in the ranges 0.1–2 μM and 2–10 μM (Figure 6a), respectively. The sensitivities were calculated based on the corresponding linear functions and showed in Figure 6b. Interestingly, we found that the EF values of different dye molecules and sensitivities of R6G on different films are consistently with the ratio of *I_G_*/*I_D_* (Figure 3b, Figure 4c, Figure 5b,d and Figure 6b), suggesting that the average size of *sp*^2^ carbon domain can significantly enhance Raman intensity on graphene substrate. The larger average size of *sp*^2^ carbon domain has a larger polarizability, which can contribute an extra local electric field to enhance Raman activity. Thus, the average size of *sp*^2^ carbon domain play a critical role in SERS activity of graphene. Owing to the larger average size of *sp*^2^ carbon domain, the GO films displayed higher SERS activity than that of CRGO films. The restoration of *sp*^2^ carbon network significantly improves SERS activity of GO-LN film. Thus, large average size of *sp*^2^ carbon domain of graphene-based substrate is needed to enhance SERS activity. Raman spectra of CV and MG molecules on GO-LN films with different concentrations are recorded in Figure 7. Raman intensities gradually increased with increasing CV, and MG concentration. A linear relationship between CV concentration and Raman intensity is observed in 0.1–3 μM and 3–15 μM (Figure 7b), respectively. The corresponding linear functions are *Y* = 246.8*X* + 7.8 (*R*^2^ = 0.980) and *Y =* 35.0*X +* 680.9 (*R*^2^ = 0.989), respectively. A good linear relationship can be obtained in the wide low concentration range from 1 to 100 μM of MG (Figure 7d), and the linear equation can be described as *Y* = 44.2*X* + 994.4 (*R*^2^ = 0.982). This proves that GO-LN was an excellent SERS substrate and the proposed SERS method is high sensitive for the determination of CV and MG in both qualitative analysis and quantitative analysis.

## 4. Conclusions

In summary, GO and CRGO films with different morphologies and structures were prepared by different simple drying methods. R6G, CV, and MG were used to identify the Raman enhancement on GO and CRGO films. The adsorption of dye molecules and surface morphologies of graphene films have little effect on Raman enhancement. The Raman enhancement factors and sensitivities of Raman probes on different films are consistent with the *I_G_*/*I_D_* ratio of graphene-based films. Higher SERS activities of GO films are observed than those of CRGO films. The results demonstrated that chemical Raman enhancement of graphene is strongly dependent on the average size of the *sp*^2^ carbon domain. Upon increasing the average size of the *sp*^2^ domain, GO-LN was shown to be an excellent SERS substrate.

## Supplementary Materials

The following are available online at http://www.mdpi.com/1996-1944/11/7/1199/s1, Figure S1: Raman spectra of R6G (10^−3^ M), CV (10^−3^ M), and MG (10^−3^ M) on glass slide substrate, Figure S2: Raman spectra of R6G molecules deposited on GO-LN films by immersing in the solution with different concentrations, Table S1: Sheet resistance of as-prepared samples characterized using four-probe resistance tester, Table S2: Raman peak positions (in cm^−1^) of R6G, CV and MG on glass slide substrate, and the relative spectral shifts and the enhancement factor (EF) values of R6G, CV and MG on the surfaces of different films. Each Raman spectrum was recorded with an integrated time of 30s, Table S3: The adsorption quantity of R6G on different substrates.
