# Supplementary material for: Strong Dependence of Surface Enhanced Raman Scattering on Structure of Graphene Oxide Film
Source: Materials (Basel). 2018 Jul 12;11(7):1199. doi: 10.3390/ma11071199 (PMC6073250; doi:10.3390/ma11071199)
Supplement: Supplementary file 1 [file materials-11-01199-s001.pdf]

materials

Supplementary information

Strong Dependence of Surface Enhanced Raman Scattering on Structure of Graphene Oxide Film

Ling Wang [1], Yan Zhang [1,*], Yongqiang Yang [2] and Jing Zhang [1]

1 School of Material Engineering, Shanghai University of Engineering Science, Shanghai 201620, China; lingwangwz@163.com (L.W.); zhangjinglyy@163.com (J.Z.)
2 Jiangsu Graphene Inspection Technology Key Laboratory, Jiangsu Province Special Equipment Safety Supervision and Inspection Institute Branch of Wuxi, Wuxi 214174, China; yqyang@wxtjy.com
* Correspondence: yanzhang@sues.edu.cn

Received: 5 June 2018; Accepted: 9 July 2018; Published: date

Electrical resistance analysis

The electrical resistance of as-prepared samples was measured by the four-point measurement method using a RTS-9 tester (4 Probes technology, China). For each pressure, five values of the applied current and the corresponding voltages were registered and the resistance was calculated.

Table S1. Sheet resistance of as-prepared samples characterized using four-probe resistance tester.

Samples	Sheet resistance (kΩ·cm)	Samples	Sheet resistance (Ω·cm)
GO-h	4.06	CRGO-h	9.70
GO-f	7.48	CRGO-f	18.20
GO-LN	2.42	CRGO-LN	8.60

Calculation of enhancement factor (EF)

The EF values are calculated according to the equation of EF= $(I_{SERS}/N_{Surf})/(I_{RS}/N_{Vol})$, where I_{SERS} and I_{RS} correspond to the peak intensities of dye molecule collected in the presence and absence of graphene-based film, and N_{Surf} and N_{Vol} are the average number of dye molecules in the scattering volume of SERS and normal Raman detections, respectively [1].

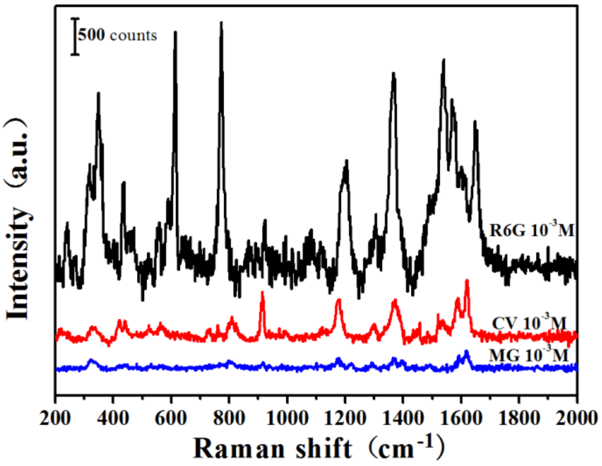

Figure S1. Raman spectra of R6G (10^{-3} M), CV (10^{-3} M), and MG (10^{-3} M) on glass slide substrate.

Table S2. Raman peak positions (in cm^{-1}) of R6G, CV and MG on glass slide substrate, and the relative spectral shifts and the enhancement factor (EF) values of R6G, CV and MG on the surfaces of different films. Each Raman spectrum was recorded with an integrated time of 30s.

Samples	–	GO-LN	GO-h	GO-f	CRGO-h	CRGO-LN	CRGO-f
R6G 1μM	614cm^{-1}	−3	−3	−3	−3	−3	−3
	EF	1.96 × 10^3	1.10 × 10^3	0.82 × 10^3	0.71 × 10^3	0.36 × 10^3	0.32 × 10^3
	773 cm^{-1}	−1	−1	−1	−1	−1	−1
	EF	1.16 × 10^3	0.62 × 10^3	0.45 × 10^3	0.43 × 10^3	0.17 × 10^3	0.14 × 10^3
	1186 cm^{-1}	−1	−1	−1	−1	−1	−1
	EF	1.81 × 10^3	1.40 × 10^3	0.99 × 10^3	0.91 × 10^3	0.38 × 10^3	0.21 × 10^3
	914 cm^{-1}	−1	−1	−1	−1	−2	−2
	EF	1.71 × 10^2	1.47 × 10^2	1.28 × 10^2	0.97 × 10^2	0.39 × 10^2	0.38 × 10^2
CV 10μM	1176 cm^{-1}	+1	+1	+1	+1	+1	+1
	EF	3.57 × 10^2	3.31 × 10^2	3.30 × 10^2	1.05 × 10^2	0.50 × 10^2	0.50 × 10^2
	1620 cm^{-1}	−4	−4	−5	−4	−4	−4
	EF	0.66 × 10^2	0.25 × 10^2	0.23 × 10^2	0.23 × 10^2	0.02 × 10^2	0.02 × 10^2
	801cm^{-1}	+5	+3	+6	+6	+5	+5
	EF	1.06 × 10^2	0.74 × 10^2	0.64 × 10^2	0.38 × 10^2	0.28 × 10^2	0.27 × 10^2
MG 100μM	1178 cm^{-1}	−1	−1	−2	−2	−3	−3
	EF	2.96 × 10^2	2.05 × 10^2	1.99 × 10^2	0.77 × 10^2	0.58 × 10^2	0.58 × 10^2
	1618 cm^{-1}	−4	−5	−5	−5	−5	−5
	EF	0.61 × 10^2	0.29 × 10^2	0.24 × 10^2	0.20 × 10^2	0.20 × 10^2	0.19 × 10^2

Adsorption experiment

Different films were immersed in 1 mL R6G solution (0.8 μM, 3 μM, 5 μM,10 μM) for 24 h, respectively. The quantities of molecules deposited on the substrates were obtained by subtracting the amount of the left R6G in the supernatant (which determined by measuring the UV absorbance of R6G solution at 527 nm) from the total R6G added.

Table S3. The adsorption quantity of R6G on different substrates.

Sample	Added R6G (×10^{-9} mol)			
(1.5 mm × 1.5 mm)	0.8	3	5	10
GO-LN	0.548	1.31	2.68	4.89
GO-h	0.477	0.99	2.23	4.20
GO-f	0.441	0.971	2.18	4.24
CRGO-h	0.394	0.871	1.28	2.63
CRGO-LN	0.453	0.93	2.22	3.52
CRGO-f	0.477	0.95	2.47	3.89

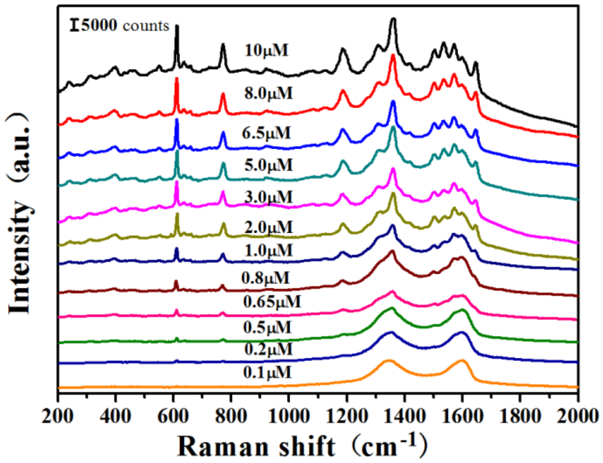

Figure S2. Raman spectra of R6G molecules deposited on GO-LN films by immersing in the solution with different concentrations.

Reference

17. Zheng, X., et al., Hydrothermal reduction of graphene oxide; effect on surface-enhanced Raman scattering. *J. Raman Spectrosc*. **2017**, 48(1), 97–103.

© 2018 by the authors. Submitted for possible open access publication under the terms and conditions of the Creative Commons Attribution (CC BY) license (http://creativecommons.org/licenses/by/4.0/).
